# Quantitative association of cerebral blood flow, relaxation times and proton density in young and middle-aged primary insomnia patients: A prospective study using three-dimensional arterial spin labeling and synthetic magnetic resonance imaging

**DOI:** 10.3389/fnins.2023.1099911

**Published:** 2023-03-21

**Authors:** Xiao-Wen Luo, Quan-Xi Li, Li-Shan Shen, Xiang Zhou, Feng-Yun Zou, Wen-Jie Tang, Ruo-Mi Guo

**Affiliations:** Department of Radiology, Third Affiliated Hospital of Sun Yat-sen University, Guangzhou, China

**Keywords:** magnetic resonance imaging, primary insomnia, quantitative imaging, cerebral blood flow, relaxation time

## Abstract

**Objectives:**

To quantitatively measure the T1 value, T2 value, proton density (PD) value, and cerebral blood flow (CBF) in young and middle-aged primary insomnia (PI) patients, and analyze the correlations between relaxation times, PD, and CBF to explore potential brain changes.

**Methods:**

Cranial magnetic resonance (MR) images of 44 PI patients and 30 healthy subjects were prospectively collected for analysis. The T1, T2, PD, and CBF values of the frontal lobe, parietal lobe, temporal lobe, and occipital lobe were independently measured using three-dimensional arterial spin labeling (3D-ASL), synthetic magnetic resonance imaging (syMRI) and a whole-brain automatic segmentation method. The differences of these imaging indices were compared between PI patients and healthy subjects. Follow-up MR images were obtained from PI patients after 6 months to compare with pre-treatment images. The Wilcoxon signed rank test and Spearman rank were used for statistical analysis.

**Results:**

Bilateral CBF asymmetry was observed in 38 patients, with significant differences in both the T2 value and CBF between the four lobes of the brain (*p* < 0.01). However, no significant difference was found in the T1 and PD values between the bilateral lobes. A negative correlation was found between CBF and T2 values in the right four lobes of patients with primary insomnia (PI). During follow-up examinations, five PI patients showed a disappearance of insomnia symptoms and a decrease in CBF in both brain lobes.

**Conclusion:**

Insomnia symptoms may be associated with high CBF, and most PI patients have higher CBF and lower T2 values in the right cerebral hemispheres. The right hemisphere appears to play a critical role in the pathophysiology of PI. The 3D-ASL and syMRI technologies can provide a quantitative imaging basis for investigating the brain conditions and changes in young and middle-aged PI patients.

## Introduction

Primary insomnia (PI) is a large class of sleep disorders characterized by poor sleep quality or short sleep duration, mainly manifested as difficulty falling asleep and staying asleep for at least 1 month (Li et al., 2016). The prevalence of insomnia in the Chinese population is 15.0%, and young people are more likely to have insomnia than older people (Cao et al., 2017), possibly related to the impact of life stress, work stress, and mental stress on sleep quality (Lee et al., 2021; Casjens et al., 2022). PI places a heavy burden on patients and health care systems and significantly impacts the quality of life of patients and the psychological, professional, and economic fields. People with PI are more likely to have mental illnesses, such as anxiety and depression, and may even be prone to suicide (Wojnar et al., 2009; Ben Simon et al., 2020; Matsui et al., 2021; Palagini et al., 2022). Therefore, understanding the neurophysiological and pathological changes in young and middle-aged PI patients is very important to treat insomnia promptly.

Despite advances in neuroimaging, the underlying neurophysiological and pathological changes in PI remain unclear. Chen L. et al. found that the trajectory intensity of the frontostriatal circuit obtained by diffusion tensor imaging (DTI), including fractional anisotropy (FA), can be used as a potential biomarker for the neuroimaging of sleep quality (Chen et al., 2021). Moghaddam, HS. et al. showed that white matter connection studies of the brain using DTI, including FA, mean diffusivity, axial diffusivity, and radial diffusivity, might provide valuable information regarding the underlying neural mechanism of PI (Sanjari et al., 2021). Yu et al. (2020) found that the severity of insomnia in PI patients directly correlates with the thicknesses of the cortex of the right orbital frontal lobe and the right fusiform area, indicating that the thickening of the cortex, especially the right cortex, maybe the neuropathological basis of PI. Rostampour et al. (2022) found that PI patients had asymmetrical, extensive white matter integrity changes in the two cerebral hemispheres, which was conducive to the diagnosis and prognosis of PI. These studies focused on the changes in brain volume and morphology in patients with insomnia. Cerebral lesions can be determined by analyzing cerebral blood flow (CBF) and relaxation times on magnetic resonance imaging (MRI) (Petitclerc et al., 2021; Krug et al., 2022; Moss et al., 2022); therefore, CBF and relaxation time may provide a new tool for the study of PI. However, the CBF and MRI relaxation times of young and middle-aged PI patients are rarely mentioned in the literature, and changes in these parameters in PI patients remain unclear.

Three-dimensional arterial spin labeling (3D-ASL) imaging uses the blood in the arteries as a tracer to generate a series of radiofrequency pulses to evaluate the CBF of a unit of brain tissue by reversing the magnetization of the blood in the artery. 3D-ASL imaging does not require an MR contrast agent, making it safer and more economical than cerebral perfusion imaging, which requires a contrast agent (Hernandez-Garcia et al., 2019). 3D-ASL technology can analyze the CBF condition of patients with insomnia (Asplund and Chee, 2013; Zhou et al., 2019). However, few studies on CBF in PI patients measured by 3D-ASL technology are currently available (Huang et al., 2022).

Synthetic MR imaging (syMRI) is a technique that uses multi-dynamic multi-echo sequences to measure the quantitative relaxation parameters of multi-contrast images, which are then used to synthesize contrast-weighted images. Compared with syMRI, traditional MRI can only obtain a specific contrast-weighted image with one scan and no quantification. syMRI simultaneously receives multiple sets of quantitative parameters with one scan: longitudinal relaxation time (T1 relaxation time), transverse relaxation time (T2 relaxation time), and proton density (PD) (Ji et al., 2020). The three quantitative values, T1, T2, and PD, are intrinsic properties of tissues that can define and identify different tissues and are independent of the scanning parameters of the MRI instrument under different field strengths (Cui et al., 2020). The syMRI technique has been applied to organs such as the brain, breast, prostate, and bladder, and the image quality is not inferior to that of traditional contrast-weighted images (Granberg et al., 2016; Cui et al., 2020; Cai et al., 2021; Matsuda et al., 2021).

The purpose of this study was to use 3D-ASL and syMRI technology (MAGnetic resonance imaging Compilation, MAGiC sequence) to quantitatively measure CBF, T1, T2, and PD values in PI patients and calculate the correlations between CBF with two relaxation times and PD, aiming to provide a noninvasive quantitative means for analyzing the brain conditions and changes in young and middle-aged PI patients.

## Materials and methods

### Study population and data collection

**Figure 1:**
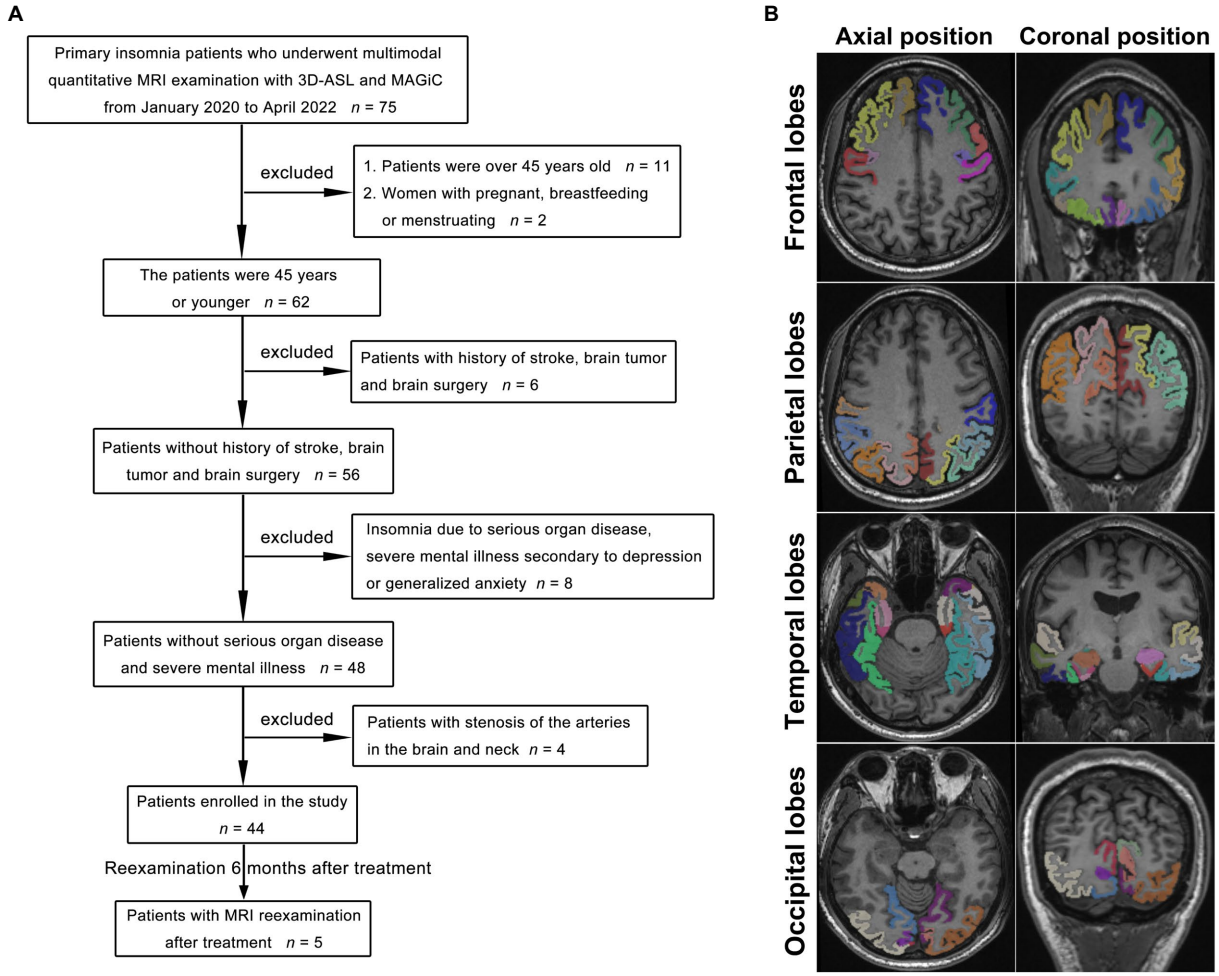
Flowchart of the PI patient selection process **(A)**. With the 3D-T1 images as a reference, the frontal lobe, parietal lobe, temporal lobe, and occipital lobe were accurately obtained by image segmentation as the ROIs for measurements **(B)**, and the different colors indicate the different gyri of the left and right lobes.

This study prospectively collected multimodal cranial MR images (3D-ASL and syMRI) from 44 young and middle-aged PI patients (20 males aged 16–45 years, average age 32.54 years; 24 females aged 15–43 years, average 31.69 years) and 30 healthy subjects (15 males aged 15 to 45 years, average age 33.55 years; 15 females aged 16–45 years, average age 32.50) who were initially admitted as outpatients from June 1, 2020, to June 30, 2022. Five PI patients underwent cranial MRI 6 months after medical treatment (Trazodone, Shenyang Funing Pharmaceutical Co., Ltd.), including 3D-ASL sequences. The diagnostic criteria for PI were based on the Diagnostic and Statistical Manual of Mental Disorders, 5th Edition (DSM-V), and were evaluated by experienced neurologists. The diagnosis of PI by a clinician met all the following criteria: (A) self-reported complaints of difficulty falling asleep, staying asleep, and/or waking up too early without being able to fall back asleep; (B) the presence of sleep difficulties at least three nights per week for at least 3 months; (C) sleep problems despite a sufficient opportunity to sleep; (D) no other, primary cause (such as obstructive sleep apnea or sleep-related movement disorder) and no causative physiological effects of any substances; and (E) no coexisting mental disorders or physical conditions that could adequately explain insomnia. The inclusion criteria for the patients (Figure 1A) were (1) meeting the abovementioned DSM-5 diagnostic criteria for PI; (2) right-handedness (avoid different effects of different handedness on CBF); (3) age ≤ 45 years; and (4) Pittsburgh Sleep Quality Index (PSQI) >5 points (Buysse et al., 1989). The inclusion criteria for healthy subjects met the second and third points of the PI patients’ inclusion criteria. For all participants, the exclusion criteria were defined as follows: (1) age > 45 years; (2) pregnant, breastfeeding, or menstruating women; (3) insomnia caused by severe organ diseases secondary to severe mental illness, such as depression or generalized anxiety; (4) history of stroke, brain tumor, or brain surgery; and (5) stenosis and variations of the arteries in the brain and neck diagnosed by MR angiography (MRA). The ethics committee approved this study ([2022] 02–005-01, [2020]02–012-01), and informed consent was obtained from all participants.

### MRI data acquisition

All MRI examinations were performed using a 3.0-T MR scanner (SIGNA Architect, GE Healthcare, Milwaukee, Wisconsin, USA) and a 48-channel phased-array head coil. Patients were still supine during the examination, and MRI examinations were arranged between 8:00 and 9:00 am to avoid the interference of different circadian rhythms on CBF. The cranial MRI scan sequences included T2-weighted fast spin–echo (FSE), T1-weighted FLAIR, T2-weighted FLAIR, three-dimensional T1-weighted imaging (3D-T1), 3D-ASL, and MAGiC. The 3D ASL scan used the 3D FSE imaging sequence with pseudo-continuous arterial spin labeling, and the post-label delay was 1.5 s; the MAGiC sequence was 2D MAGiC, and the scan used the FSE imaging sequence, with two echoes and four delayed acquisitions. The MR sequence parameters are shown in Table 1. The multi-delay multi-echo (MDME) data were reconstructed and analyzed using MAGiC software on a 64-bit Advantage workstation (GE Healthcare).

**Table 1:** MRI acquisition parameters.

	T2 FSE	T1 FLAIR	T2 FLAIR	MRA	3D-T1	3D-ASL	MAGiC
TR(ms)	5,514	1750	8,500	20	7.7	4,664	4,000
TE (ms)	103	11.5	89.3	2.5	3.1	53.6	13.9&90.2
TI (ms)	NA	720	2,411	NA	450	1,500(PLD)	28
FOV (cm)	24 × 24	24 × 24	24 × 24	24 × 24	25.6 × 25.6	24 × 24	24 × 24
Slice thickness (mm)	5	5	5	1	1	4	4
Interslice gap (mm)	1	1	1	0	0	0	1
Acquisition matrix	352 × 352	320 × 256	260 × 260	256 × 256	256 × 256	245 × 245	320 × 256
NEX	1	2	1	1	1	3	1
Bandwidth (kHz)	83.3	50	41.67	20	31.25	62.5	50
Flip Angle	110	111	110	20	12	111	NA
Acceleration	2	2	2	2	2	NA	2

TR, repetition time; TE, echo time; TI, inversion time; PLD, post label delay; FOV, field of view; NEX, number of excitations; NA, not measured; MRA, MR angiography.

### Image measurements

With the 3D-T1 images as a reference, the quantitative T1-, T2-, and PD-volume fraction mapping (T1 map, T2 map, and PD map) images were first calculated from the SyMRI data using vendor-provided postprocessing software (SyntheticMR, v11.2.2, GE Healthcare, USA). The original image of CBF was imported by Volume Viewer (v15.0, GE Healthcare, USA) software postprocessing. Then, the linear transformation matrix between T1 map images and 3D-T1 images and nonlinear warped images between 3D-T1 images and T1W template images in Montreal Neurological Institute (MNI) space were obtained using Advanced Normalization Tools (ANTs). T1 map, T2 map, and PD map images were then normalized to the standard MNI template by applying the above linear transformation matrix and nonlinear warped images. The same process was used to normalize the CBF images to the MNI template, and the linear transformation was calculated between the control image of 3D-ASL and the 3D-T1 image. Subsequently, the mean values of the warped images were measured within different brain regions. These brain regions were defined on the International Consortium for Brain Mapping 152 template (Figure 1B). Image analysis and data measurement were performed by two radiologists with extensive diagnostic experience (working for more than 10 years). In addition, every value of cranial MR images of five PI patients who were reexamined after treatment was compared before and after treatment. The units of the T1 value and T2 value are milliseconds (ms), the PD value is given in units of pu (represents the hydrogen proton density per voxel, which is a percentage unit), and the CBF value is given in mL/100 g/min (which indicates how many milliliters of blood flow through 100 g of brain tissue per minute). The symmetry between the measured values of the left and right lobes indicated a lack of significant difference, whereas asymmetry was defined as a significant difference between lobes.

### Statistical analyses

All data were statistically analyzed using Prism 9 software (GraphPad Software Inc., San Diego, CA, USA). All data from each patient ‘s initial visit that were not normally distributed and presented as the median (first quartile - third quartile). The data for the five reexamined patients were presented as the mean value. The differences in T1, T2, PD, and CBF values between the bilateral frontal, parietal, temporal, and occipital lobes of PI patients or healthy subjects were measured and analyzed with the Wilcoxon signed rank test with Bonferroni ‘s correction. The correlation coefficients between the CBF, two relaxation times, and PD of the frontal, parietal, temporal, and occipital lobes were calculated by Spearman rank to explore the brain conditions and changes. A scatter plot between the CBF value and the PSQI value was generated, and the correlation coefficients were also calculated by Spearman rank between the CBF and PSQI values. *p* < 0.05 was considered statistically significant.

## Results

### Quantitative measurement of T1, T2, PD, and CBF values in both cerebral hemispheres of the PI patients

**Figure 2:**
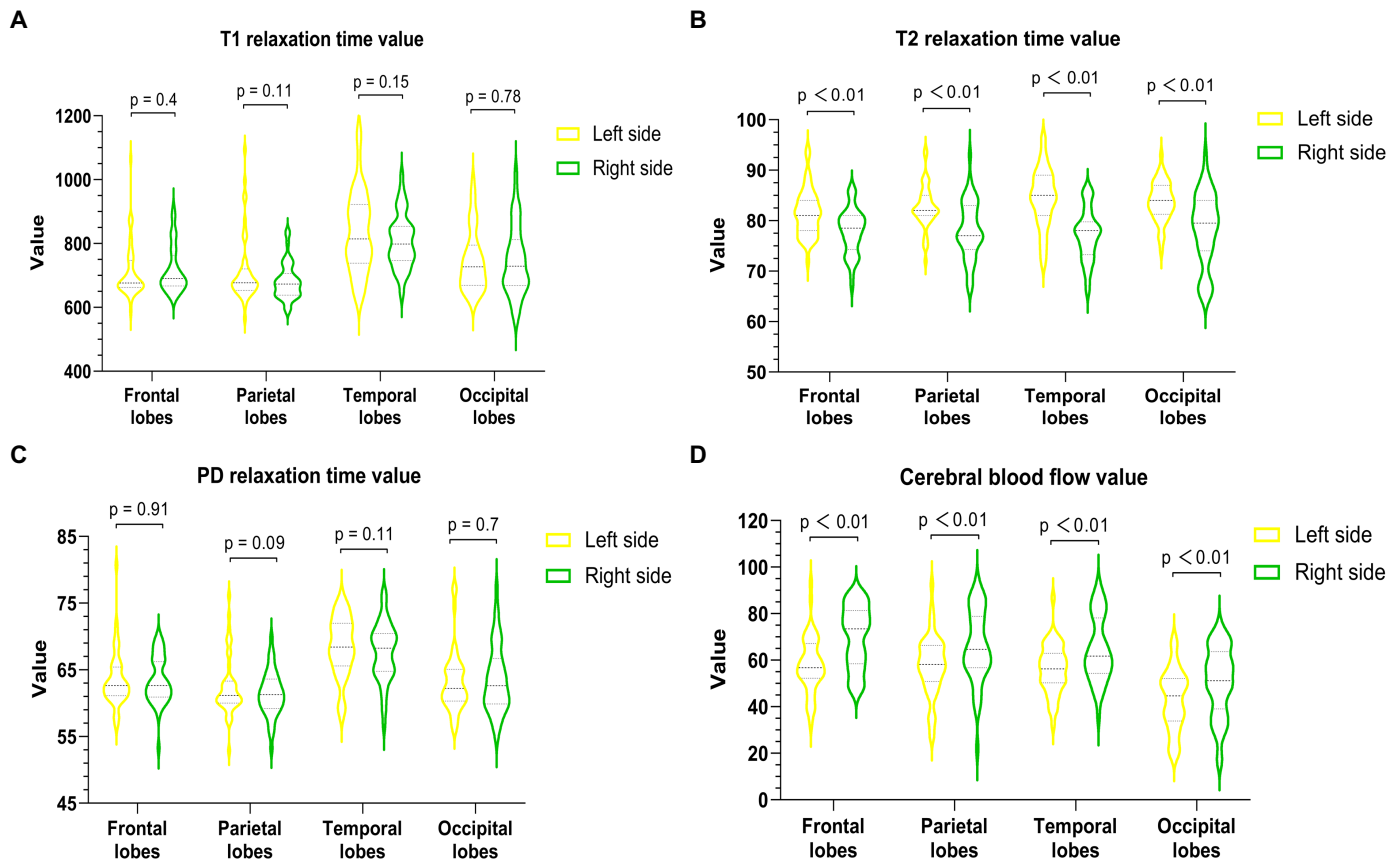
Quantitative measurement of T1 **(A)**, T2 **(B)**, PD **(C)**, and CBF **(D)** of the bilateral frontal lobe, parietal lobe, temporal lobe, and occipital lobe in PI patients. The T2 value of each right lobe was significantly lower than that of the corresponding left lobe (*p* < 0.01), and the CBF value of each right lobe was markedly higher than that of the corresponding left lobe (*p* < 0.01).

All images and data were clear and usable. The T1, T2, and PD values of the frontal, parietal, temporal, and occipital lobes were measured (Table 2). The T1 values did not significantly differ between the bilateral frontal, parietal, temporal, and occipital lobes (*p* values: 0.4, 0.11, 0.15, and 0.78, Figure 2A). The T2 values in the right frontal, parietal, temporal, and occipital lobes were significantly lower than the corresponding T2 values on the left (*p* < 0.01, Figure 2B). The PD values of the bilateral frontal, parietal, temporal, and occipital lobes were not significantly different (*p* values: 0.91, 0.09, 0.11, and 0.7, Figure 2C). Among the pseudocolor ASL in 44 PI patients, 38 (86.36%) patients had asymmetric bilateral CBF (Figure 4), and among the MRA in 44 PI patients, all the patients had normal cerebrovascular (Figure 4). Specifically, the CBF value of the right hemisphere was larger than that of the left hemisphere, and only six (13.64%) patients had symmetric bilateral CBF. The CBF values of the frontal, parietal, temporal, and occipital lobes were measured (Table 2). The CBF values of the right frontal, parietal, temporal, and occipital lobes were significantly higher than the corresponding values of the left lobe (*p* < 0.01, Figure 2D).

**Table 2:** The comparison of T1, T2, PD, and CBF values between healthy subjects and PI patients.

		T1 value (ms)	T2 value (ms)	PD value (pu)	CBF value (mL/100 g/min)
Left frontal lobe	Healthy	679 (660–738)	82 (75–84)	62 (60–65)	57 (53–66)
	PI patients	678 (663–739)	82 (78–84)	63 (61–65)	57 (52–67)
	*p* value	0.387	0.162	0.229	0.112
Right frontal lobe	Healthy	688 (664–742)	83 (74–87)	62 (62–68)	58 (54–68)
	PI patients	690 (668–750)	79 (75–81)	63 (61–66)	74 (60–81)
	*p* value	0.322	**0.006**	0.158	**0.001**
Left parietal lobe	Healthy	676 (631–704)	82 (78–86)	62 (60–64)	57 (52–67)
	PI patients	678 (653–717)	82 (81–85)	61 (60–63)	58 (51–66)
	*p* value	0.361	0.129	0.144	0.108
Right parietal lobe	Healthy	674 (635–693)	82 (74–86)	62 (60–64)	57 (52–69)
	PI patients	673 (639–703)	78 (75–83)	62 (59–63)	65 (58–79)
	*p* value	0.308	**0.009**	0.173	**0.005**
Left temporal lobe	Healthy	812 (716–984)	86 (78–89)	69 (67–71)	56 (50–65)
	PI patients	815 (746–922)	85 (81–89)	68 (66–72)	56 (50–63)
	*p* value	0.287	0.142	0.201	0.118
Right temporal lobe	Healthy	805 (722–980)	87 (81–89)	68 (66–71)	55 (53–66)
	PI patients	799 (750–851)	78 (74–79)	68 (65–70)	62 (55–78)
	*p* value	0.258	**0.007**	0.191	**0.004**
Left occipital lobe	Healthy	726 (666–789)	85 (81–89)	62 (60–66)	44 (35–52)
	PI patients	727 (672–793)	84 (82–87)	62 (60–65)	45 (34–52)
	*p* value	0.357	0.162	0.227	0.111
Right occipital lobe	Healthy	728 (663–791)	86 (82–88)	63 (60–66)	43 (35–52)
	PI patients	729 (673–798)	80 (74–84)	63 (60–66)	51 (40–62)
	*p* value	0.346	**0.008**	0.243	**0.006**

PI, primary insomnia. All data that were not normally distributed and presented as the median (first quartile – third quartile). The units of the T1 value and T2 value are milliseconds (ms), the PD value is given in units of pu (represents the hydrogen proton density per voxel, which is a percentage unit), and the CBF value is given in mL/100 g/min (which indicates how many milliliters of blood flow through 100 g of brain tissue per minute). The bold values showed statistical difference (*p* < 0.05).

### Scatter plot between the CBF value and the PSQI value

**Figure 3:**
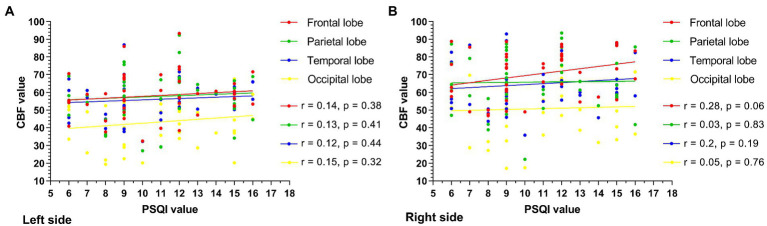
The scatter plot between the CBF value and the PSQI value is shown. The CBF values were not correlated with PSQI values in the left **(A)**, right **(B)** frontal, parietal, temporal, and occipital lobes, and all the *p* values of the left and right cerebral lobes exceeded 0.05.

The PSQI values were collected and ranged from 6 to 16. The scatter plots between the CBF value and the PSQI value are shown (Figure 3). The CBF values were not correlated with PSQI values in the left (Figure 3A) and right (Figure 3B) frontal, parietal, temporal, and occipital lobes (left frontal lobe: *r* = 0.14, *p* = 0.38; right frontal lobe: *r* = 0.28, *p* = 0.06; left parietal lobe: *r* = 0.13, *p* = 0.41; right parietal lobe: *r* = 0.03, *p* = 0.83; left temporal lobe: *r* = 0.12, *p* = 0.44; right temporal lobe: *r* = 0.2, *p* = 0.19; left occipital lobe: *r* = 0.15, *p* = 0.32; right occipital lobe: *r* = 0.05, *p* = 0.76).

**Figure 4:**
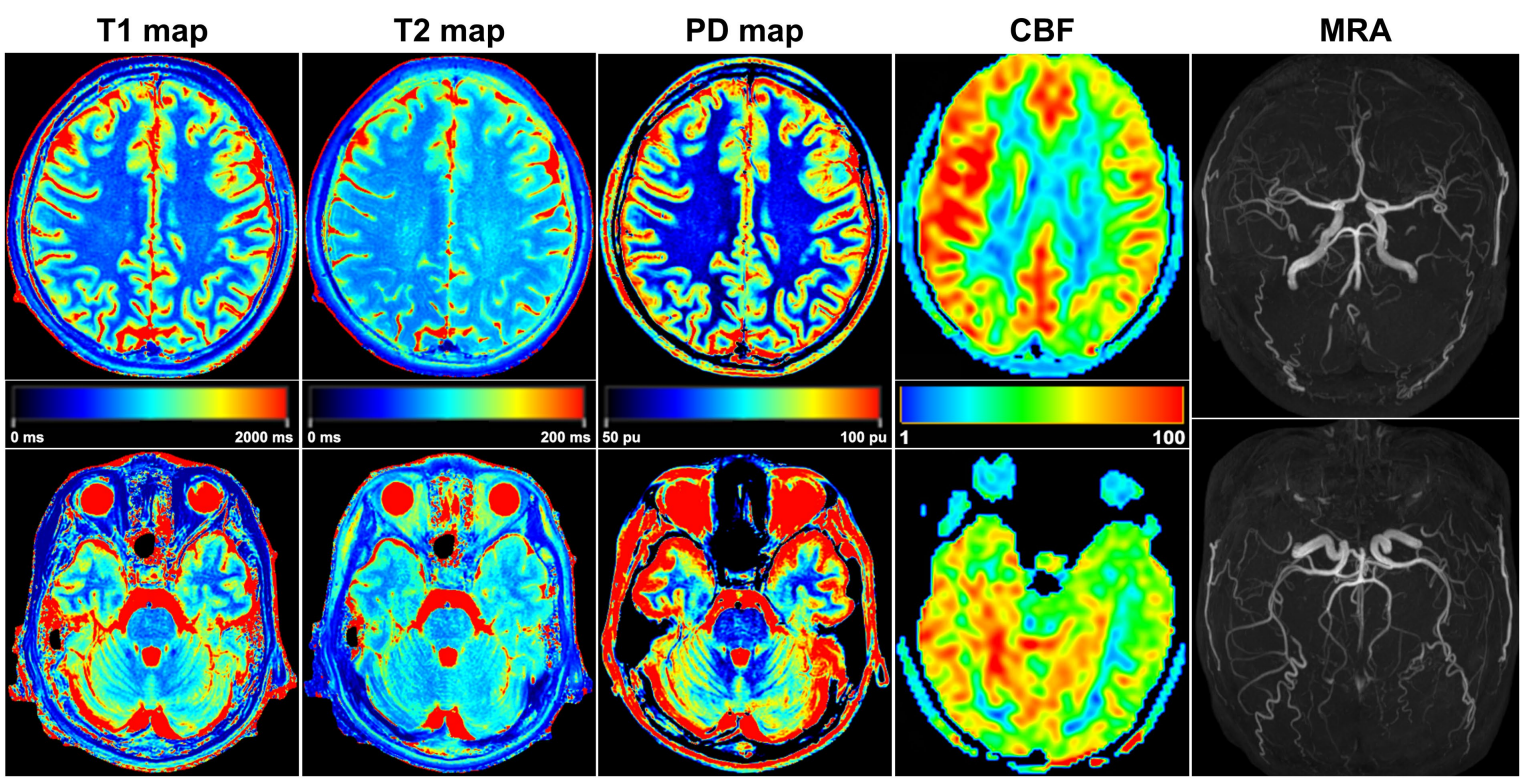
A 32-year-old female PI patient. Five sets of pseudocolor images of the T1 map, T2 map, PD map, CBF, and MRA were obtained. In the T2 map, the T2 value was lower in the right lobes than in the corresponding left lobes. The CBF value of each right lobe was higher than that on the left side, and the bilateral CBF was asymmetrical. MRA showed that the shape and course of each cerebral artery were normal, and no arterial stenosis or variation was found.

### Correlation analysis of T1, T2, PD, and CBF values in both cerebral hemispheres of the PI patients

**Figure 5:**
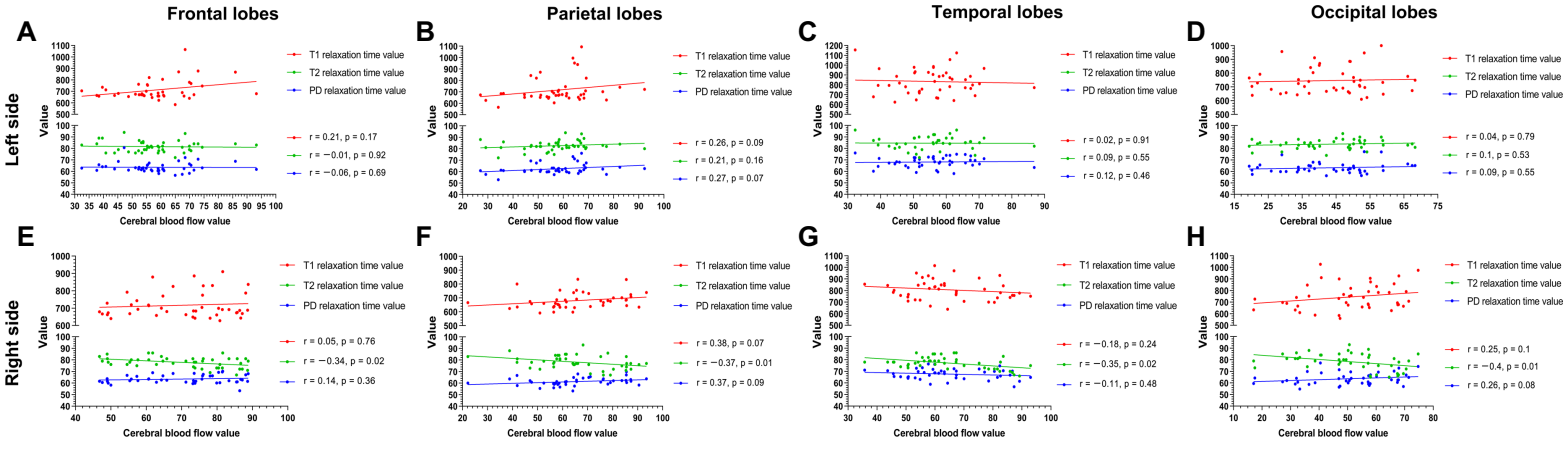
The measured values of PI patients in each group **(A–H)**. The CBF and T1 values were not correlated between the bilateral frontal, parietal, temporal, and occipital lobes. The CBF and T2 values were negatively correlated in the right lobe (right frontal lobe *p* = 0.02; right parietal lobe *p* = 0.01; right temporal lobe *p* = 0.02; right parietal lobe *p* = 0.01) **(E–H)** but were not correlated in any left lobe **(A–D)**. CBF was not correlated with PD in the left and right frontal, parietal, temporal, and occipital lobes.

Among the measured values of PI patients in each group (Figure 5), the CBF and T1 values were not correlated between the bilateral frontal, parietal, temporal, and occipital lobes. The CBF and T2 values of the right lobes showed negative correlations (right frontal lobe: *r* = −0.34, *p* = 0.02; right parietal lobe: *r* = −0.37, *p* = 0.01; right temporal lobe: *r* = −0.35, *p* = 0.02; right occipital lobe: *r* = −0.4, *p* = 0.01), with no correlation in the left lobes. CBF was not correlated with PD in the left and right frontal, parietal, temporal, and occipital lobes.

### Quantitative measurement of T1, T2, PD, and CBF values in both cerebral hemispheres of the healthy subjects

The T1 and T2 values of the frontal lobes, parietal lobes, temporal lobes, and occipital lobes were measured (Table 2). The T1 and T2 values did not significantly differ between the left and right cerebral hemispheres (T1 *p* values: 0.38, 0.14, 0.68, and 0.79; T2 p values: 0.42, 0.27, 0.52, and 0.62). Furthermore, the PD values of the bilateral frontal, parietal, temporal, and occipital lobes were not significantly different (p values: 0.74, 0.58, 0.43, and 0.65, Table 2). All healthy subjects (100.00%) had symmetric bilateral CBF. The CBF values of the bilateral frontal, parietal, temporal, and occipital lobes were not significantly different (p values: 0.58, 0.42, 0.47, and 0.62, Table 2).

### Comparison of T1, T2, PD, and CBF values between healthy subjects and PI patients

The T2 values in the right frontal, parietal, temporal, and occipital lobes of PI patients were significantly lower than the corresponding T2 values on the right lobes of healthy subjects (*p* < 0.01). Furthermore, the CBF values of the right frontal, parietal, temporal, and occipital lobes of PI patients were significantly higher than the corresponding values of the right lobes of healthy subjects (p < 0.01). In addition, the T1 and PD values of the bilateral frontal, parietal, temporal, and occipital lobes did not significantly differ between healthy subjects and PI patients (Table 2).

### Reexamination of five PI patients

**Figure 6:**
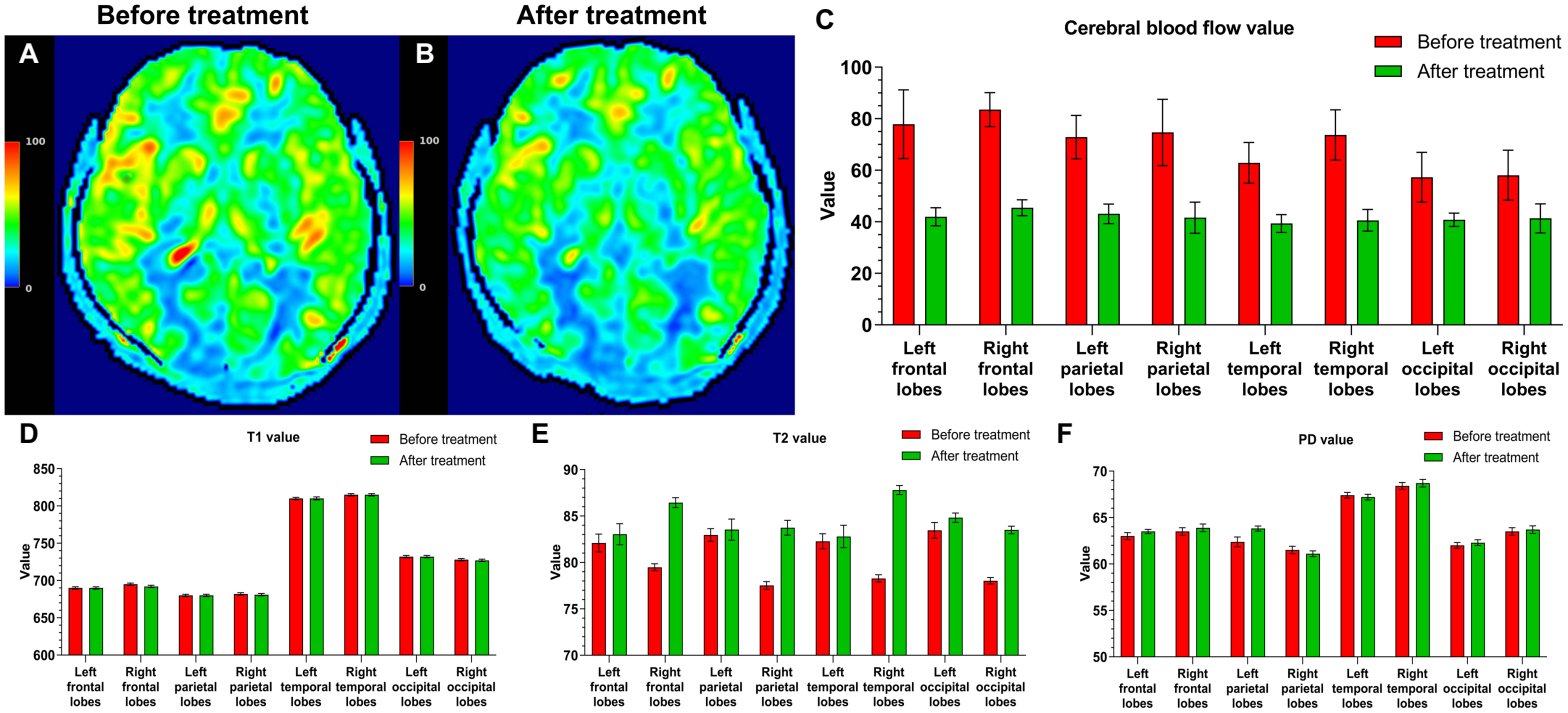
Five PI patients were reexamined 6 months after treatment. Cranial MRI showed that in a CBF pseudocolor image, the signal in the high-intensity area **(A)** of the initial diagnosis was reduced **(B)**. The CBF values after treatment were also lower than those before treatment **(C)**. The T2 values of all five patients were higher than those before treatment **(E)**, and the T1 and PD values did not change after and before treatment **(D,F)**.

Five PI patients whose insomnia symptoms had disappeared were reexamined 6 months after treatment. The clinical data for five patients before and after treatment are shown in Table 3. The CBF values of all five patients were lower after treatment than before treatment (Figures 6A–C). The T2 values of all five patients were higher after treatment than before treatment, but the T1 and PD values did not change after treatment (Figures 6D–F). Based on their wishes, the remaining 39 patients did not undergo a repeated MRI examination due to improved insomnia symptoms.

**Table 3:** The clinical data of five PI patients with reexamination.

	Gender	Age (y)	Insomnia severity index		Pittsburgh sleep quality index	
			Before treatment	After treatment	Before treatment	After treatment
Patient 1	Male	32	18	5	12	5
Patient 2	Female	29	21	7	15	5
Patient 3	Male	31	16	5	9	4
Patient 4	Male	33	20	7	15	6
Patient 5	Female	31	18	6	12	4

PI, primary insomnia.

## Discussion

This study found that the bilateral CBF values of most PI patients were asymmetrical and higher in each right lobe than in the corresponding left lobe. At the same time, the T2 value of each right lobe was significantly lower than that of the left lobe. Moreover, the CBF values of the right lobes of PI patients were significantly higher than those of the corresponding right lobes of healthy subjects, and the T2 values in the right lobes of PI patients were significantly lower than the corresponding T2 values in the right lobes of healthy subjects. CBF and T2 values in PI patients were negatively correlated in each right lobe.

During insomnia, increased brain thinking activity causes neuronal activation (Galbiati et al., 2021). An increase in neuronal activity leads to an increase in CBF. When the overall brain activity is increased (for example, during hyperthyroidism), CBF is increased; when brain activity is inhibited (for example, in a coma), CBF is reduced (Iadecola, 2017). Some studies have confirmed that CBF increases during rapid-eye-movement sleep and dreaming, especially in the right temporal–parietal region (Meyer et al., 1987). These results are consistent with this study’s increasing CBF values observed in the right brain lobes. We speculate that PI patients have increased activity in the right hemisphere, causing neuronal excitement and a CBF increase in the right lobe (Li et al., 2017; Yan et al., 2018; Zhu et al., 2021). Furthermore, the thicker cortex in some areas of the right hemisphere may be a potential cause of higher CBF in the right hemisphere (Yu et al., 2020), which might be because larger brain regions contain more large vessels and have a superior ASL signal-to-noise ratio (SNR) than smaller regions (Várkuti et al., 2011). When we reexamined five PI patients 6 months after treatment, their insomnia symptoms disappeared, and the CBF of the bilateral brain lobes also decreased. This result suggests that insomnia symptoms are possibly related to a high CBF.

An interesting aspect of our study is the negative correlation between increased CBF and decreased T2 value. These correlations imply a link between increased deoxyhemoglobin (increased oxygen consumption) leads to reduced T2 and increased CBF, similar to previously reported conjecture (Salehi et al., 2023). After 6 months of treatment, the T2 value of five PI patients also increased as the blood flow decreased. These findings explain the excellent correlation between CBF and T2 on the right side of each brain lobe in PI patients. Therefore, the T2 value can be used to determine the cranial condition of patients with insomnia. However, CBF and PSQI values did not correlate in this study, perhaps because the sample size was insufficient or the age range of enrollment was limited; these factors will be further examined in future studies.

Here, the MAGiC sequence of syMRI technology yielded a quantitative map of T1, T2, and PD *via* one scan in a shorter time (4 min). The T1, T2, and PD values allow absolute measurements of the characteristics of each lobe of the brain, providing a basis for the quantitative diagnosis of PI from cerebral morphology (Kim et al., 2022). 3D-ASL can stably and quantitatively measure the CBF of the brain without the use of a contrast agent, and the examination is safe and reproducible (Mahroo et al., 2021). The brain segmentation technique accurately drew the ROI of each lobe, and the two sequences MAGiC and 3D-ASL were used to accurately measure each lobe, providing a quantitative imaging basis for exploring the neurophysiological and pathological changes in PI.

The first limitation of our study was that all PI patients were ≤ 45 years old. The age range could be expanded in the future, allowing for a broader range of studies. Another limitation was the acquisition of the 2D characteristics of the MAGiC sequence. Since our center did not have access to the 3D MAGiC sequence of syMRI technology, we used 2D MAGiC sequence acquisition. The signal of the 2D sequence is inferior to that of the 3D sequence; therefore, the 3D MAGiC sequence should be used whenever possible in the future. The third limitation was that only five PI patients had MRI examinations during reexamination, and the lack of a placebo control group limited the interpretation of the posttreatment results. In future studies, more reexamined patients and a placebo control group can hopefully undergo MRI examinations to deeply understand the brain function changes in PI patients before and after treatment. The fourth limitation was the ROI measurement bias due to partial volume effect of synMRI and ASL images. Since both the syMRI and ASL images were acquired with low spatial resolution in either slice or all directions, while the ROIs were defined in high-resolution T1 template, the measurement bias cannot avoid. In future, high-resolution 3D mapping techniques may provide more accurate measurement.

## Conclusion

Insomnia symptoms are potentially associated with high CBF, and most PI patients have higher CBF and lower T2 values in the right cerebral hemispheres. The CBF and T2 values were negatively correlated in each right lobe. The right cerebral hemisphere plays an essential role in the pathophysiological mechanism of PI. Therefore, 3D-ASL and syMRI technology can provide a quantitative imaging basis for exploring brain conditions and changes in young and middle-aged PI patients.

## Data availability statement

The original contributions presented in the study are included in the article/supplementary material, further inquiries can be directed to the corresponding author.

## Ethics statement

This study was approved by the Institutional Research Ethics Committee of the Third Affiliated Hospital of Sun Yat-Sen University ([2022]02-005-01 and [2020]02-012-01). Written informed consent to participate in this study was provided by the participants’ legal guardian/next of kin.

## Author contributions

X-WL and R-MG data collection, data analysis, and manuscript writing. Q-XL, X-WL, F-YZ, and XZ data collection. L-SS, X-WL, XZ, W-JT, and R-MG data analysis and manuscript editing. R-MG project development, data analysis, and manuscript editing. All authors contributed to the article and approved the submitted version.

## Funding

This study was supported by grants from the National Natural Science Foundation of China (No. 81801757), Guangdong Basic and Applied Basic Research Foundation (No. 2022A1515010369, 2023A1515010256), and Guangzhou Science and Technology Plan (No. 202201020421).

## Conflict of interest

The authors declare that the research was conducted in the absence of any commercial or financial relationships that could be construed as a potential conflict of interest.

## Publisher’s note

All claims expressed in this article are solely those of the authors and do not necessarily represent those of their affiliated organizations, or those of the publisher, the editors and the reviewers. Any product that may be evaluated in this article, or claim that may be made by its manufacturer, is not guaranteed or endorsed by the publisher.
